# Azurophil Granule Proteins Constitute the Major Mycobactericidal Proteins in Human Neutrophils and Enhance the Killing of Mycobacteria in Macrophages

**DOI:** 10.1371/journal.pone.0050345

**Published:** 2012-12-14

**Authors:** Prajna Jena, Soumitra Mohanty, Tirthankar Mohanty, Stephanie Kallert, Matthias Morgelin, Thomas Lindstrøm, Niels Borregaard, Steffen Stenger, Avinash Sonawane, Ole E. Sørensen

**Affiliations:** 1 School of Biotechnology, Campus-11, KIIT University, Bhubaneswar, Orissa, India; 2 Division of Infection Medicine, Department of Clinical Sciences, Lund University, Lund, Sweden; 3 Institute for Medical Microbiology and Hygiene, University of Ulm, Ulm, Germany; 4 Department of Infectious Disease Immunology, Statens Serum Institute, Copenhagen, Denmark; 5 Department of Hematology, University of Copenhagen, Copenhagen, Denmark; University Medical Center Utrecht, The Netherlands

## Abstract

Pathogenic mycobacteria reside in, and are in turn controlled by, macrophages. However, emerging data suggest that neutrophils also play a critical role in innate immunity to tuberculosis, presumably by their different antibacterial granule proteins. In this study, we purified neutrophil azurophil and specific granules and systematically analyzed the antimycobacterial activity of some purified azurophil and specific granule proteins against *M. smegmatis, M. bovis*-BCG and *M. tuberculosis* H37Rv. Using gel overlay and colony forming unit assays we showed that the defensin-depleted azurophil granule proteins (AZP) were more active against mycobacteria compared to other granule proteins and cytosolic proteins. The proteins showing antimycobacterial activity were identified by MALDI-TOF mass spectrometry. Electron microscopic studies demonstrate that the AZP disintegrate bacterial cell membrane resulting in killing of mycobacteria. Exogenous addition of AZP to murine macrophage RAW 264.7, THP-1 and peripheral blood monocyte-derived macrophages significantly reduced the intracellular survival of mycobacteria without exhibiting cytotoxic activity on macrophages. Immunofluorescence studies showed that macrophages actively endocytose neutrophil granular proteins. Treatment with AZP resulted in increase in co-localization of BCG containing phagosomes with lysosomes but not in increase of autophagy. These data demonstrate that neutrophil azurophil proteins may play an important role in controlling intracellular survival of mycobacteria in macrophages.

## Introduction

The immune responses to *Mycobacterium tuberculosis* (*Mtb*) are complex and poorly understood. The early host response to *Mtb* infection involves primarily resident alveolar macrophages and infiltrated neutrophils [1]. Macrophages mount a complex immune response to the invading *Mtb*, but the pathogen is able to chronically reside and proliferate inside the host for extended period of time by manipulating microbicidal functions such as inhibition of phago-lysosome fusion, production of reactive oxygen species (ROS) and nitric oxide, and by rendering macrophages non-responsive to interferon gamma (IFN-γ) [2].

Although, macrophages have long been regarded as the key phagocytic cells in mycobacterial infection [3], [4], [5], there are increasing evidences showing protective role of neutrophils in tuberculosis [6], [7] but the exact role of neutrophils in innate protection against mycobacteria is not fully understood. Evidences for the role of neutrophils in innate immunity to tuberculosis (TB) include the observations that during the early stages of *Mtb* infection polymorphonuclear leukocytes (PMN) migrate and accumulate at the site of infection [8], [9], the risk for TB infection diminish with increased neutrophil count, and killing of *M. bovis* BCG in a whole blood was significantly impaired by neutrophil depletion [10] and in neutrophil serine proteases cathepsin G and neutrophil elastase deficient mice [11]. Furthermore, innate immune responses to *Mtb* in RAG-deficient mice showed a compensatory function for neutrophils in controlling the bacterial burden in the absence of IFN-γ from T cells [12]. The role of neutrophils in TB is conceivable from the study showing that no granuloma formation was observed in PMN depleted mice up to 60 days of post infection with *Mtb* [13].

Human neutrophil kill mycobacteria through oxygen-independent mechanisms, since neutrophils from patients with chronic granulomatous disease are just as effective in killing mycobacteria as normal neutrophils [14]. However, only few data regarding bactericidal activities of neutrophil granular proteins toward mycobacteria are available. Human neutrophil peptides (HNPs) [15], [16], [17] have been shown active against mycobacteria. The ability of macrophages to kill mycobacteria has been found increased by acquiring the proteins from other cells, for example, by delivery of granulysin from cytotoxic granules of T-lymphocytes [18] or myeloperoxidase from neutrophils [19]. It has recently been shown that when macrophages phagocytose apoptotic neutrophils, this leads to reduction in viability of intracellular *Mtb* [20].

In this study we modified and developed a gel overlay assay that was described previously [21] to identify the neutrophil granule proteins with mycobactericidal activity. The intracellular growth characteristics of *M. smegmatis* and BCG have been well characterized in RAW 264.7 [22] and THP-1 cells [23], respectively. For this reason we chose these two models to evaluate the role of neutrophil granule proteins in mycobacteria killing. We found that azurophil granules are the granule type containing the proteins with most killing activity against mycobacteria. Electron microscopic studies revealed that azurophil granule proteins kill mycobacteria by disintegrating the cell morphology. Treatment of macrophages with azurophil granule proteins increased intracellular killing of mycobacteria and enhanced phago-lysosome fusion, but did not result in enhanced autophagy.

## Results

### Whole neutrophils-mediate killing of mycobacteria

**Figure 1:**
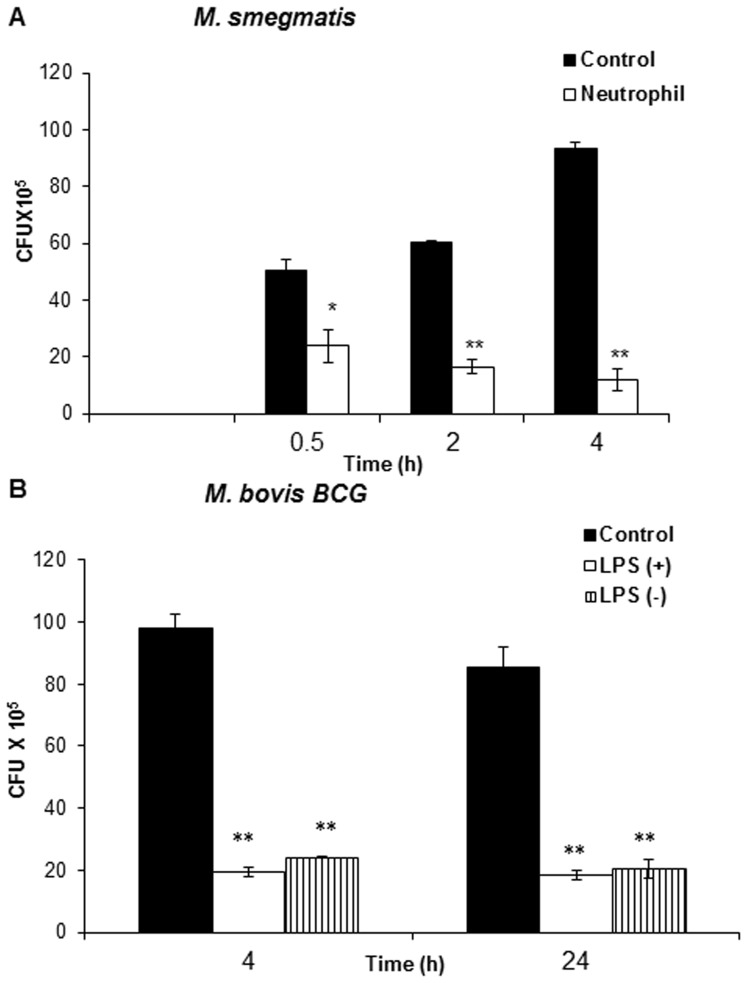
Survival of *M. smegmatis* and *M. bovis* BCG in LPS-stimulated and unstimulated whole neutrophils. 4–9×10^5^/ml *M. smegmatis* (**A**) and BCG (**B**) were incubated with LPS stimulated neutrophils (+) and unstimulated neutrophils (−) for indicated time points. Bacterial survival was determined by CFU assay. Medium containing bacteria alone was used as control. Data shown are from one representative experiment of three individual experiments. Experiments were performed in triplicates; mean ± SD are shown; Significance was referred as ** for P<0.0001.

We initiated our studies on the role of neutrophils in mycobacterial infection by characterizing the kinetics of mycobacterial survival in the presence of neutrophils. Exponentially-grown *M. smegmatis* and *M. bovis* BCG representing fast (generation time ∼3 h) and slow-growing (generation time ∼19 h) mycobacteria, respectively, were incubated with LPS-stimulated neutrophils and the number of colony forming unit (CFU) was analyzed by harvesting bacteria at different time points by plating serial dilutions and surviving colonies were counted after 3 days and 3 weeks for *M. smegmatis* and BCG, respectively. As shown in Figure 1A and 1B, LPS-primed neutrophils significantly reduced the number of viable mycobacteria, with approximately 80% (P<0.0001) of *M. smegmatis* and BCG population being killed after 30 min and 4 h, respectively. 24 hours incubation of BCG with neutrophils did not give rise to further significant reduction in bacterial viability (Figure 1B). As mentioned before because of their short and long generation time, the viability of *M. smegmatis* and BCG was determined after approximately one-generation time. Previous studies have shown that LPS stimulation augments the antimicrobial activity of neutrophils [24]. To test this, we compared the susceptibility of *M. smegmatis* and BCG to non-stimulated neutrophils. No statistically significant differences in the bacterial killing were observed between non-stimulated and LPS-stimulated neutrophils (Figure 1B).

### Neutrophil azurophil granule proteins are highly active against mycobacteria

**Figure 2:**
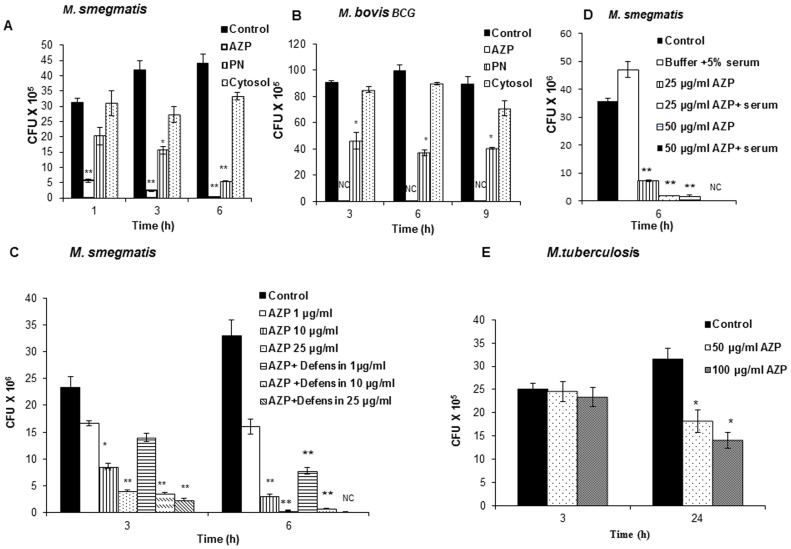
*M. smegmatis*, *M. bovis-*BCG and *M. tuberculosis* H37Rv survival following incubation with neutrophil granule proteins. 4–9×10^5^/ml *M. smegmatis* (**A**) and BCG (**B**) were incubated with 50 µg/ml each AZP, PN and cytosolic proteins for indicated time points and the survival of mycobacteria was determined by CFU assay. Bacteria containing Tris-glucose buffer was used as control. NC indicates no bacterial growth. (**C**) *M. smegmatis* was grown in defensin-depleted (AZP) and defensin containing AZP (AZP+defensin) and the bacterial viability was determined after 3 and 6 h of incubation. CFU was determined by plating the bacteria in 7H10 middlebrook agar plates. Bacteria grown in Tris-glucose buffer was used as control. (**D**) *M. smegmatis* was grown in Tris glucose buffer containing 5% human serum and without serum (control) in the presence of 25 and 50 µg/ml AZP for 6 h. The survival of bacteria was determined by plating CFU. (**E**) *M. tuberculosis* survival following incubation with 50 and 100 µg/ml AZP for 3 and 24 hours. Data shown are from one representative experiment of three individual experiments. Experiments were performed in triplicates; mean ± SD are shown; Significance was referred as ** for P<0.0001.

Neutrophils are endowed with antimicrobial proteins present in various types of granules. Subcellular fractionation of disrupted neutrophils was performed on a two-layer Percoll density gradient, which separates peroxidase-positive (azurophil) granules from peroxidase-negative granules (specific and gelatinase granules). The anti-mycobacterial activity of α-defensins, which are present in azurophil granules, has already been reported [25], [26], [27]. To identify new antimycobacterial proteins, α-defensins were depleted from azurophil granules (referred to as AZP in the following text) by cation exchange chromatography as previously described [28]. To evaluate the relative mycobactericidal activities, we compared the microbicidal activities of proteins from defensin-depleted azurophil granules (AZP), peroxidase negative (PN) granules and cytosolic proteins against *M. smegmatis* and BCG. Exponentially grown bacteria were incubated with 50 µg/ml proteins. As shown in Figure 2A, the mycobactericidal activity of AZP was substantially more than that of PN granule proteins against *M. smegmatis* and BCG, with more than 80% and 95% (P<0.0001) of the *M. smegmatis* were being killed after 1 h and 6 h of incubation, respectively; whereas in case of BCG complete killing was observed after 3 h of incubation (P<0.0001; Figure 2B). To determine the minimum concentration of AZP required for BCG killing, the bacteria were incubated with different doses of AZP ranging from 5–50 µg/ml for 3 h. About 65–70% of BCG killing was observed with 25 µg/ml proteins (supplementary Figure S1). The antimycobacterial activity of peroxidase-negative granule proteins was moderate compared to that of AZP pproximately 60% and 80% killing of *M. smegmatis* was observed after 3 h and 6 h incubation, respectively; whereas BCG was found to be less susceptible (50% and 65%) over the period of the study. Cytosolic proteins did not display any major mycobactericidal activity.

To determine the contribution of α-defensins to the mycobactericidal properties of azurophil granules, we compared the microbicidal activities of AZP alone and α-defensin containing AZP. Killing of *M. smegmatis* by AZP and defensin-containing AZP occurred in a dose-dependent manner (P<0.0001; Figure 2C). Incubation of defensin-containing AZP showed a more prominent killing of *M. smegmatis* compared with AZP alone demonstrating a role of defensins in killing of mycobacteria with azurophil granule proteins. Complete killing of *M. smegmatis* was observed in AZP containing 50 µg/ml α-defensins (data not shown).

The activity of many antimicrobial proteins is often inhibited by complex body fluids such as serum, saliva, or sputum [29], [30]. Therefore, we investigated the antimycobacterial activity of AZP in the presence of serum. Serum did not inhibit but actually increased the antimycobacterial activity of these granule proteins (P<0.0001; Figure 2D).

As AZP were found to be more active against *M. smegmatis* and BCG in comparison to PN and cytosolic proteins, we compared the susceptibility of *M. tuberculosis* H37Rv to AZP only. In comparison to *M. smegmatis* and BCG, *M. tuberculosis* was found to be more resistant such that about 55% killing was observed with 100 µg/ml after 24 h of incubation (Figure 2E). No significant killing was observed at lower concentrations (below 50 µg/ml) of AZP (data not shown).

### Subcellular fractionation of AZP and PN granules revealed numerous proteins exhibiting anti-mycobacterial activity

**Figure 3:**
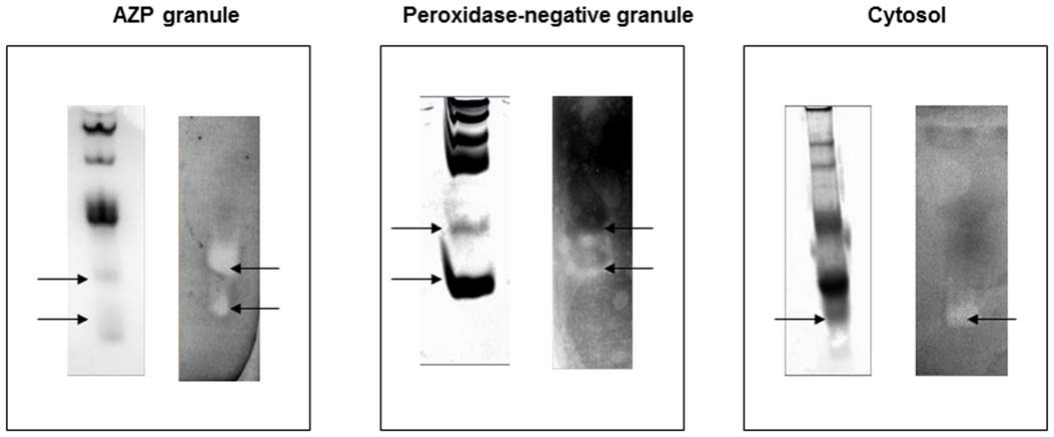
Gel overlay assay of AZP, PN and cytosol proteins. Azurophile (AZP), peroxidase-negative (PN) and cytosol proteins were run on AU-PAGE, Coomassie stained (left) and analyzed for antibacterial activity against *M. smegmatis*. Formation of clearing zone indicates the antimycobacterial activity (right).

To identify the granule and cytosolic proteins with antimycobacterial activity, AZP, PN and cytosolic proteins were electrophoresed on AU-PAGE gel and protein bands with antimycobacterial activity were identified by an antimycobacterial gel overlay assay with *M. smegmatis*. Distinct clearing zones were found in samples with AZP, PN and cytosolic proteins (Figure 3).

**Figure 4:**
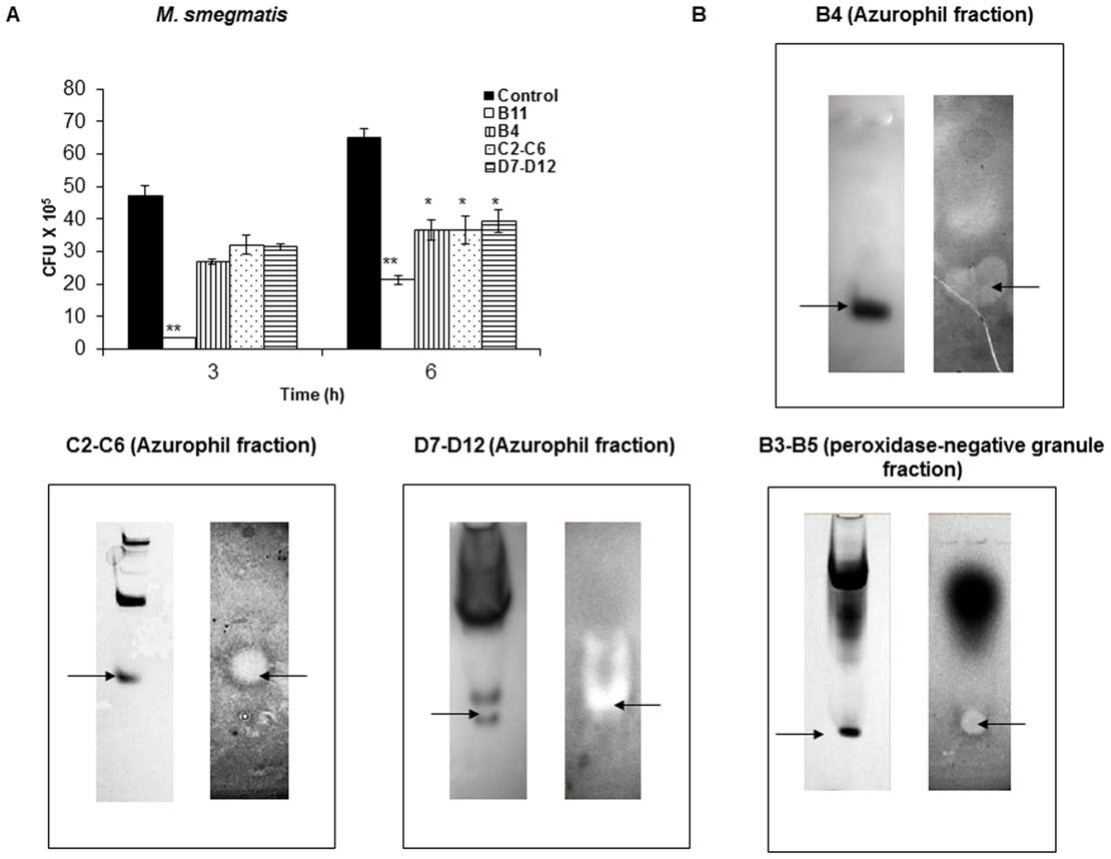
Antimycobacterial activity of AZP and PN granule proteins determined by CFU and gel overlay assays. (**A**) *M. smegmatis* (4–9×10^5^/ml) was incubated with 50 µg/ml AZP fractions for 3 and 6 h, and CFU assay was performed to check the antimycobacterial effect of the protein fractions. Bacteria with buffer only served as control. Data are mean ± SD of three independent experiments. Significance was referred as ** for P<0.0001 and * for P<0.005. (**B**) Gel overlay assay of AZP and PN granular protein fractions against *M. smegmatis*. The granule proteins stained with Coomassie brilliant blue (left) and the clearing zone formed by the protein (right) shows the anti-mycobacterial activity.

To further investigate the specific granule proteins responsible for antimycobacterial activity, AZP and PN granule proteins were further fractionated by cation exchange chromatography (Supplementary Figure S2). Since cytosolic proteins did not show significant killing activity (Figure 2A and 2B) and that single protein band showed clearing zone (Figure 3) we did not fractionate these proteins. The obtained AZP and PN granule fractions were subsequently tested for their *in vitro* antibacterial activity against *M. smegmatis*. From AZP, five peaks *viz.* fraction B11 (eluted at 15 min from another batch of AZP), B4 (eluted at 19 min), C2–C6 (eluted at 22 min), C9–C12 (eluted at 26 min), and D7–D12 (eluted at 30 min), presented high level of antimicrobial activity against *M. smegmatis*, with more than 50% growth inhibition was observed after 6 h incubation (P value<0.0005; Figure 4A). From PN granules one specific peak containing fraction B3–B5 (eluted at 18 min) showed prominent antimycobacterial activity. The results obtained under *in vitro* condition were further confirmed by gel overlay assay. As shown in Figure 4B, all the fractions from AZP and PN granules showed prominent inhibition zones.

To identify the proteins with antimycobacterial activity, the corresponding protein bands showing zone of inhibition were extracted from gels, digested with trypsin and analyzed by MALDI-TOF-MS. All protein spots could be identified and assigned to proteins deduced from human genome. Table 1 summarizes the results of the mass spectrometric analysis. The table lists the names of the proteins, their location in neutrophil, and molecular masses calculated from the genome data. Our preparation of neutrophils will contain eosinophils and therefore we identified eosinophil cationic protein (ECP) as a granule protein with mycobactericidal activity in line with a previous report [31].

**Table 1:** Identification of AZP, PN and cytosol proteins by MALDI-TOF mass spectrometry.

Protein Fraction	Protein Name	Location	Molecular Weight(KDa)
AZP Granule Proteins:			
B4	Lysozyme	Azurophil	14.4
B11	Elastase	Azurophil	29
C2–C6	ECP	Eosinophil primary matrix	18.3
D7–D12	Azurocodin/HBP	Azurophil	37
PN Granule Proteins:			
Whole PN granule *	Lactoferrin	PN	80
B3–B5	Lactoferrin	PN	14.4
Cytosolic Proteins:			
Whole Cytosol proteins *	Calgranulin	Cytosol	18

* These proteins were identified from total PN and cytosolic proteins (cf. Figure 3).

### Electron microscopy of AZP treated mycobacteria

**Figure 5:**
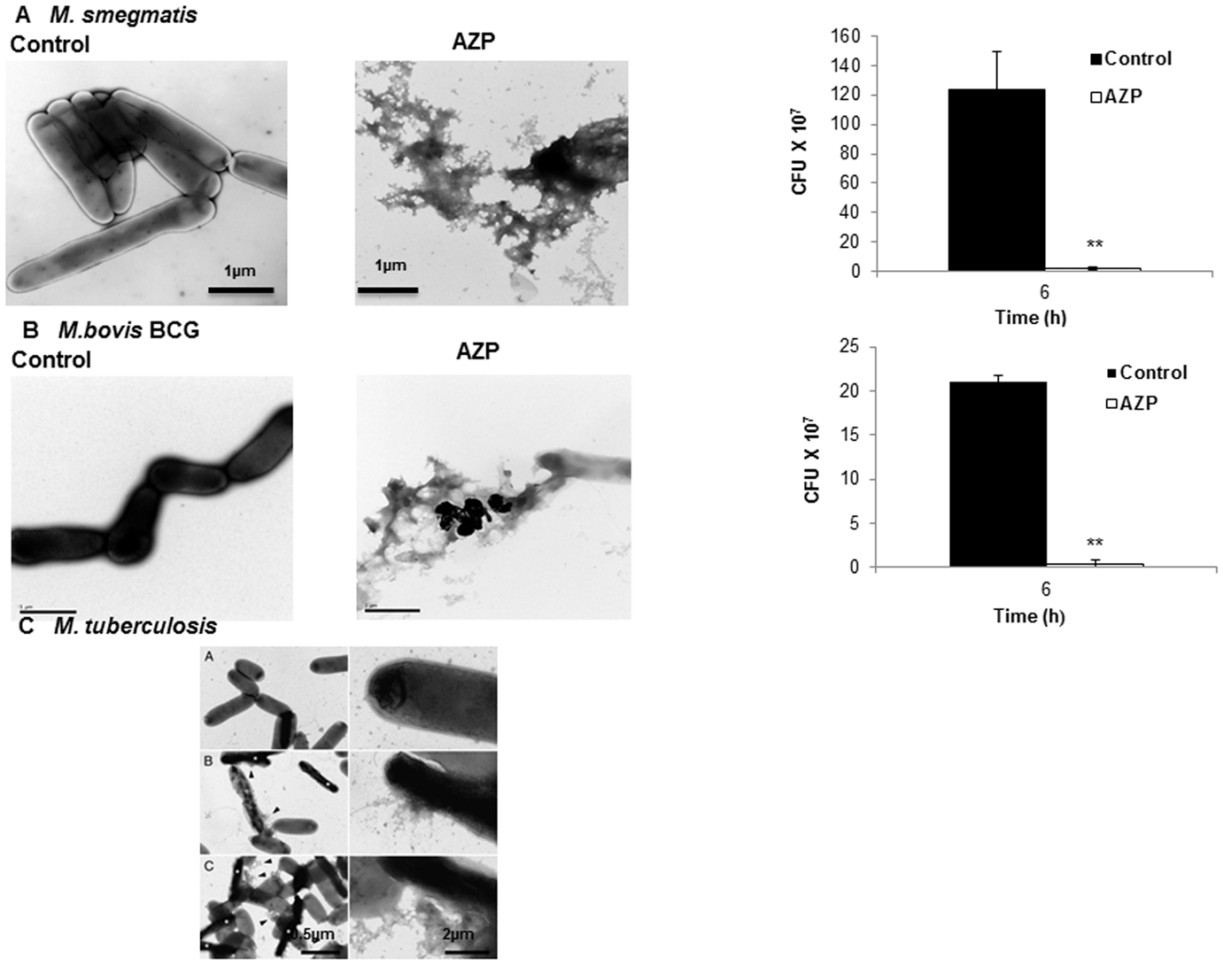
Electron micrograph of AZP treated mycobacteria. 2–10×10^7^/ml *M. smegmatis* (**A**) and *M. bovis* BCG (**B**) were incubated with or without AZP (50 µg/ml) for 6 h, *M.tuberculosis* (**C**) without (a) and with AZP (100 µg/ml) for 6 h (b) and 12 h (c) and visualized by transmission electron microscopy. The survival of *M. smegmatis* and *M. bovis* BCG as determined by CFU assay is shown in inset corresponds to the similar experimental conditions followed for electron microscopy experiment. Scale bars: 1 µm for *M.smegmatis* and *M.bovis* BCG. Scale bars: 2 µm (left panel) and 0.5 µm (right panel) for *M.tuberculosis*.

To further examine the mycobacterial killing by AZP, *M. smegmatis* and BCG bacteria were incubated with AZP (50 µg/ml) for 6 h. Mycobacterial cell wall morphology were analyzed by electron microscopy. In case of *M. tuberculosis* the electron microscopic studies were performed at 100 µg/ml AZP concentration after 12 h incubation. As shown in Figure 2E, *M*. *tuberculosis* showed more resistance to AZP action and require more exposure time to achieve effective killing. Bacteria grown in Tris-glucose buffer served as control. Treatment with AZP completely disrupted the cell structure and the cells appeared as aggregate, whereas untreated cell remained intact (Figure 5A and 5B). In case of *M.tuberculosis*, damage of cell wall was visible after 6 h treatment, whereas complete disruption of cell structure was noticed after 12 h of incubation with AZP (Figure 5C). Control experiments showed more than 95% killing of the *M. smegmatis* and BCG by AZP in the samples used for electron microscopy.

### Exogeneous addition of AZP increase intracellular killing of mycobacteria by macrophages

Phagosomes containing non-pathogenic mycobacteria readily fuse with lysosomes leading to elimination of the bacilli, whereas pathogenic mycobacteria survive inside the macrophages by preventing the phago-lysosome fusion [32]. However, phagosomes with killed pathogenic mycobacteria readily undergo phago-lysosome fusion. Accordingly, we investigated whether exogenous addition of AZP could access these phagosomes and affect the intracellular survival of mycobacteria.

**Figure 6:**
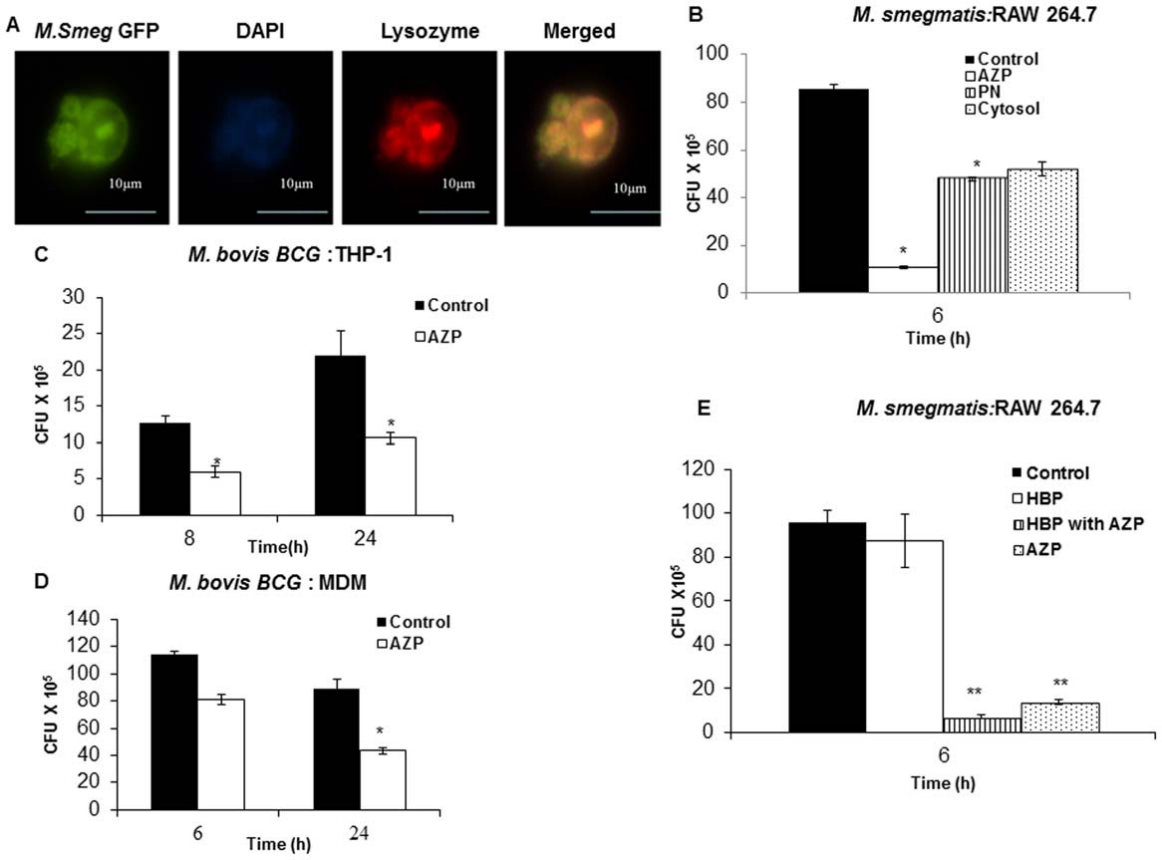
Localization of lysozyme and *M. smegmatis* in THP-1 cells. Intracellular survival of mycobacteria in AZP, PN and cytosolic protein treated macrophages. (**A**) THP-1 cells were infected with *M. smegmatis*-GFP and then treated with 100 µg/ml alexa fluor 594 labelled lysozyme for 6 h. The localization of lysozyme and *M. smegmatis* was analyzed using fluorescence microscopy. (**B**) RAW264.7 macrophages were infected with *M. smegmatis* and treated with 50 µg/ml AZP, PN and cytosolic proteins for 6 h. THP-1 (**C**) and monocyte-derived macrophages (**D**) were infected with *M. bovis* BCG and then treated with 50 µg/ml AZP. Cells were lysed at the indicated time points and bacterial intracellular survival was determined by CFU assay. (**E**) Survival of *M. smegmatis* in RAW cells treated with 25 µg/ml HBP and 50 µg/ml AZP for 6 h. Macrophages infected with bacteria alone were used as a control. Experiments were performed in triplicates. Mean ± SD are shown. Significantly different from the control: ** P<0.0001 and * P<0.005.

First we performed immunofluorescence studies using FITC-conjugated lysozyme, one of the antimycobacterial proteins identified in this study, to evaluate the internalization of exogenously added lysozyme by macrophages. Our microscopic studies showed uptake of lysozyme by macrophages into vesicular-like structures (Supplementary Figure S3). To determine whether the AZP proteins co-localize with mycobacteria, macrophages were infected with *M. smegmatis* (MOI 10) followed by treatment with alexa fluor labelled lysozyme (100 µg/ml) for 6 h. The results showed that *M. smegmatis* co-localizes with lysozyme inside the macrophages (Figure 6A). Then we tested intracellular survival of *M. smegmatis* in AZP, PN and cytosolic protein treated RAW264.7 cells. These cells were first infected with *M. smegmatis* for 2 h followed by a treatment with 50 µg/ml AZP, PN and cytosolic proteins. Untreated cells were used as a control. We saw two different patterns of bacterial killing. A significant reduction (∼85%; P value<0.0001) in *M. smegmatis* survival was observed in AZP treated cells 6 h post-infection, whereas PN and cytosolic protein treated macrophages only showed moderate increased intracellular killing as compared to non-treated cells (p<0.005; Figure 6B). This demonstrated that AZP were most potent in stimulating the intracellular killing of mycobacteria. For this reason the intracellular survival of BCG was determined only in AZP treated THP-1 and MDM cells. These cells were first infected with BCG for 3 h and then treated with 50 µg/ml of AZP. In both THP-1 and MDM cells treatment with AZP increased intracellular killing of BCG (P value<0.005; Figure 6C and 6D).

Heparin-binding protein (HBP) display bactericidal activity and boost bacterial phagocytosis by human and murine macrophages [33]. Consequently, we investigated whether addition of HBP to AZP would further potentiate the killing efficiency of macrophages. A moderate decrease in intracellular bacterial burden was observed in macrophages treated with a mixture of HBP and AZP as compared to AZP alone. Treatment with HBP alone did not show any statistically significant increased killing activity of macrophages (P value<0.0001; Figure 6E).

### Purified neutrophil granular proteins increase the intracellular killing of mycobacteria

**Figure 7:**
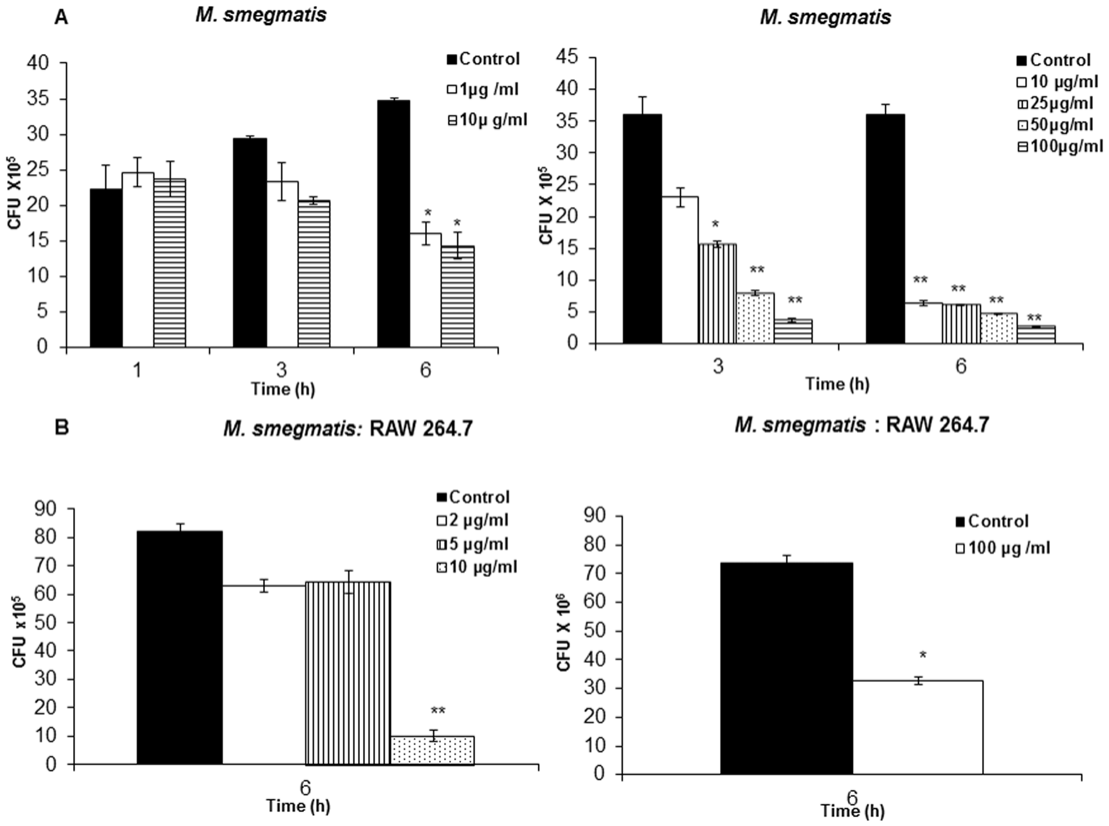
Survival of *M. smegmatis* in the presence of purified elastase and lysozyme. (**A**) *M. smegmatis* was incubated with different concentrations of elastase and lysozyme for the indicated time points and the bacterial survival was determined by CFU assay. (**B**) Intracellular survival of *M. smegmatis* in RAW264.7 cells treated with different concentrations of elastase and and 100 µg/ml of lysozyme for 6 h. Macrophages infected with *M. smegmatis* alone were used as control. Data shown represents the mean ± SD of three independent experiments. Significance was referred as ** for P<0.0001 and * for P<0.005 versus control conditions.

To investigate whether the increase in intracellular killing of mycobacteria stimulated by AZP was a result of a single protein or the aggregate of AZP, we investigated the activity of purified elastase and lysozyme both identified in the gel overlay assay. Both the proteins were active against *M. smegmatis* by CFU assay, where both proteins caused substantial dose-dependent killing of *M. smegmatis* with lysozyme being more active than elastase (P value<0.0001; Figure 7A). Treatment of RAW 264.7 cells with both elastase and lysozyme increased the intracellular killing of the mycobacteria. However, in this case elastase was more potent than lysozyme. Elastase limited the growth of *M. smegmatis* in a dose-dependent manner, while lysozyme showed significant inhibition of bacterial growth only at 100 µg/ml dose after 6 h of treatment (P value<0.005; Figure 7B) and no significant killing was observed at lower doses of lysozyme (data not shown). These data demonstrated that individual azurophil granule proteins stimulated the intracellular killing of mycobacteria in RAW 264.7 cells. This potency of the effect was, furthermore, not strictly correlated to the direct antimycobacterial effect found *in vitro* by CFU assay.

### The increased intracellular killing stimulated by AZP is not due to increased cytotoxicity

**Figure 8:**
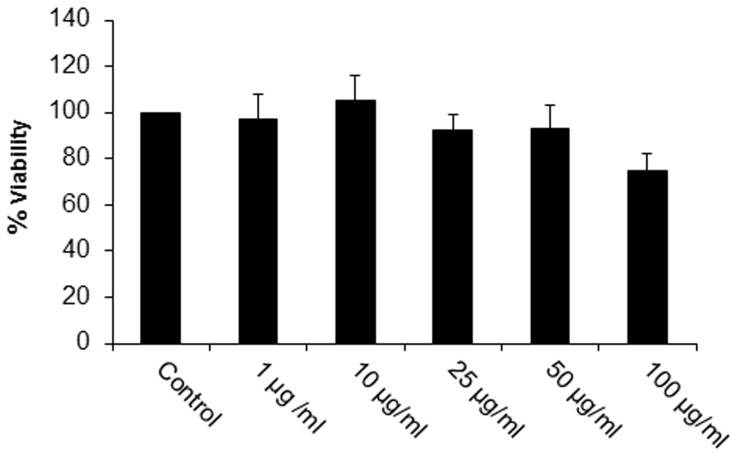
Cytotoxic activity of AZP on RAW 264.7 cells. Macrophages were treated with different concentrations of AZP for 24 h. Cells viability was determined by MTT assay. Experiments were performed in triplicates; mean ± SD are shown. Percentage of cell viability was determined from three independent experiments.

To investigate whether the decrease in intracellular bacterial survival was due to cytotoxic effect of AZP on macrophages, we tested the cytotoxicity in AZP treated macrophages by MTT assay. No increase in cytotoxicity was found after treatment with AZP (Figure 8). Similar results were obtained with THP-1 cells (data not shown).

### Treatment with AZP increases co-localization of mycobacteria-containing phagosomes with lysosomes in macrophages

**Figure 9:**
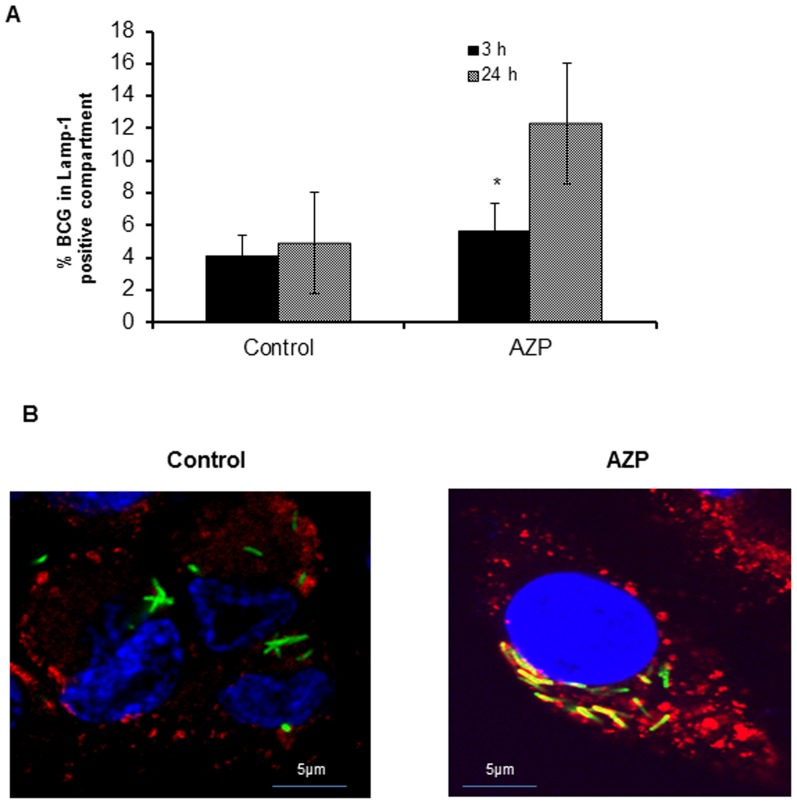
Quantification of co-localization of BCG containing phagosomes with lysosomes in AZP treated THP-1 cells. (**A**) Co-localization of BCG containing phagosomes with lysosomes in AZP treated and untreated cells were quantified microscopically by acquisition of LAMP-1 marker of late endosome/lysosome by BCG phagosomes. THP-1 cells infected with *M. bovis* BCG-GFP (green) were exposed to AZP for 24 h and stained with antibody against LAMP-1 (red) followed by secondary antibody Alexa Fluor 594 and DAPI (blue) for nuclei staining. (**B**) A representative cell showing co-localization of BCG-containing phagosome with LAMP-1 marker. THP- cells infected with BCG-GFP only were used as control. Data shown are the average of 3 individual experiments. Significance was referred as* for *p*<0.005.

As mentioned above, phagosomes containing non-pathogenic mycobacteria readily fuse with lysosomes leading to bacterial elimination, whereas pathogenic mycobacteria as well as BCG survive inside the macrophages by preventing the phago-lysosome fusion. However, phagosomes with killed pathogenic mycobacteria readily fuse with lysosomes. Also as shown above, we observed a co-localization of *M. smegmatis* with lysozyme in THP-1 cells. Consequently, we tested whether AZP increased localization of BCG-containing phagosomes with lysosomes. THP-1 cells were infected with *M. bovis*-BCG- GFP (MOI 1∶10) and treated with 50 µg/ml of AZP for 24 h. Co-localization of BCG-GFP containing phagosomes with lysosomes was quantified microscopically by evaluating the acquisition of LAMP-1, a membrane marker of late endosomes/lysosomes. As shown in Figure 9A no significant increase in the localization of BCG-containing phagosomes with LAMP-1 was observed in AZP treated and untreated THP-1 cells after 3 h post infection. In contrast, treatment with AZP significantly increased the fraction of mycobacterial phagosomes labeled for this marker at 24 h after infection (P value<0.005). A representative cell showing co-localization of BCG phagosome with LAMP-1 is shown in Figure 9B. Thus, treatment of cells with AZP increased the co-localization of BCG containing phagosomes with lysosomes in THP-1 cells.

### Azurophil granule proteins increase intracellular killing of mycobacteria by an autophagy-independent mechanism

**Figure 10:**
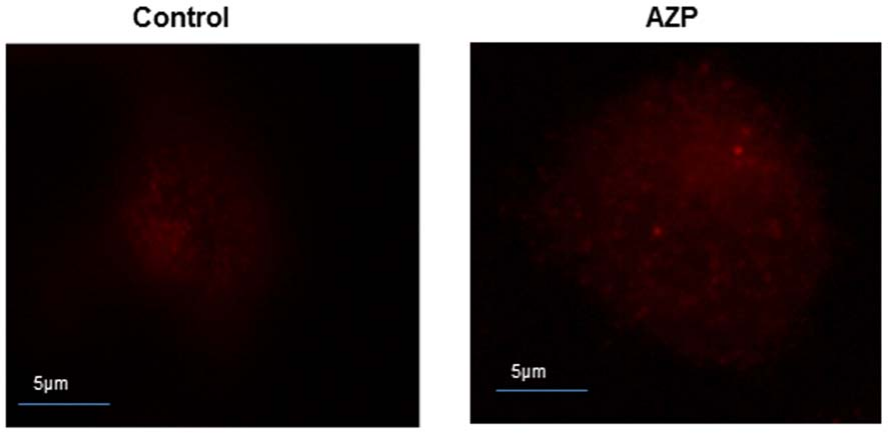
Induction of autophagy in AZP treated macrophages. THP-1 cells were treated with AZP for 24 h and stained with antibodies against LC3 to visualize the formation of auto-phagosomes.

Previous studies demonstrated that the host antimicrobial peptides induce autophagy in human monocytes through activation of autophagy-related genes *Beclin-1* and *Atg5* and also induce co-localization of mycobacteria phagosomes with autophagosomes, which mediate the killing of intracellular *M. tuberculosis* [34]. Since we observed increase in intracellular bacterial killing and co-localization of BCG containing phagosomes with LAMP-1 compartment after treatment with AZP, we investigated whether this was due to activation of autophagy in THP-1 cells. First we tested the transcripts of the mammalian autophagy-related genes *Beclin-1* and *Atg5* by real-time PCR after treatment with AZP. No significant increase in *Beclin-1* and *Atg5* expression levels were observed at 6 h and 24 h after treatment with AZP (data not shown). We also determined the expression of microtubule-associated protein 1 light chain 3 (LC3) vesicles, which reflects the number of autophagosomes, in azurophil granule proteins stimulated THP-1 cells by immunofluorescence and Western blotting. As shown in Figure 10, treatment with AZP did not cause increase in endogenous LC3 aggregates after 24 h. These results indicate that azurophil granule proteins increase intracellular killing of mycobacteria by an autophagy-independent mechanism.

## Discussion

Although many data have demonstrated a key function of macrophages in immunity against mycobacteria, very few data regarding bactericidal activities of neutrophils toward mycobacteria are available. Recent studies have shown that neutrophils may play a more important role in defense against mycobacterial infection than previously thought. Circulating neutrophils become activated and are recruited to lungs early in *Mtb* infection. Their role in host defense against *Mtb* is supported by studies showing that depletion of circulating neutrophils before i.v. challenge with *M.tb* compromises the immune response against mycobacterial infection [35] and induce granuloma formation [13]. Though neutrophils use both oxidative and non-oxidative microbicidal mechanisms to kill bacteria, human neutrophils seem to kill mycobacteria mainly through oxygen-independent mechanisms, since neutrophils from patients with chronic granulomatous disease are just as effective in killing mycobacteria as normal neutrophils [14].

Neutrophils contain many antimicrobial proteins stored in different granules, but no systematic and comparative studies have been done to identify the importance of different granules or granule proteins in killing of mycobacteria. In this study, we have purified neutrophil granules, identified the specific granular proteins responsible for the killing of pathogenic and non-pathogenic mycobacteria, and studied the mechanism by which addition of these granule proteins facilitated the intracellular killing of mycobacteria by macrophages. No significant differences in the killing kinetics of mycobacteria were observed in LPS-stimulated and non-stimulated neutrophils indicating that mycobacteria-associated protein(s) itself are sufficient to cause activation of neutrophils. This observation extends earlier finding that mycobacterial antigens can activate neutrophils [36] and that mycobacteria can induce the release of cellular constituents during interaction with human neutrophils [37]. We found that AZP exhibit more potent mycobactericidal activity than to peroxidase-negative granule proteins and cytosolic proteins. Many bactericidal proteins are localized in the azurophil granules-for example azurocidin [38], defensins [39], lysozyme and serine proteases such as cathepsin G, proteinase 3, and elastase [40]. These proteins have been shown to possess antimicrobial activity against a diverse group of microorganisms. Gel overlay assay of PN granules showed inhibition of *M. smegmatis* growth by lactoferrin. Though the role of lactoferrin in enhancing the efficacy of BCG vaccine has been reported earlier [41], the direct antimycobacterial activity of lactoferrin has not yet been described in more detail. Lactoferrin being an iron-binding protein may inhibit mycobacterial growth by limiting the availability of iron, which is an essential component for growth and survival of mycobacteria. Together with AZP and PN granule proteins, calgranulin, the cationic calcium binding protein, was found to be the only cytosolic protein having antimycobacterial activity. Calgranulin shows sequence homology with cystic fibrosis antigen and the brain S 100 calcium binding protein, which have specific zinc binding sites [42]. As shown previously, the zinc-binding property of calprotectin may be responsible for the antimycobacterial activity, by depleting zinc required for the growth of mycobacteria [43]. The increased susceptibility of mycobacteria to AZP in the presence of α-defensins showed that defensins are key effector molecule for human resistance to *Mtb*. It has been proved that α-defensins such as human neutrophil peptides 1–3 can kill *Mtb* [15]. Altogether, these results provide evidence that neutrophils harbor a diverse arsenal of cytotoxic components against mycobacteria.

Pathogenic *Mtb* was found to be less susceptible to AZP than non-pathogenic strains. The decreased susceptibility could probably be due to differences in the cell wall architecture of pathogenic and non-pathogenic mycobacteria. It has been shown that the multilayered cell wall of pathogenic *Mtb* contains less number of transporters [44] and that the lipoarabinomannan is capped with mannose. These along with other cell wall components insulate the bacteria from its environment and limit the binding and transport of drugs. Interestingly, pathogenic mycobacteria block the fusion of azurophil granules with the neutrophil phagosome [45]. Our data demonstrate that by blocking fusion of azurophil granules with the phagosome, mycobacteria are not exposed to the most potent mycobactericidal neutrophil granule proteins.

We have previously found that addition of the antimicrobial peptide, LL-37, to macrophages enhances the mycobactericidal activity of these cells [17]. Consequently, we tested whether addition of AZP would enhance the bactericidal activity of macrophages. Indeed, addition of AZP to infected macrophages enhanced the intracellular killing of mycobacteria.

Autophagic pathways have been shown to enhance intra-phagosomal killing of *M. tuberculosis* [46] and other studies [34] have shown that cathelicidin is required for 1,25D3-induced autophagy activation in monocytes and macrophages and thereby increase killing of intracellular mycobacteria. However, AZP did not stimulate autophagy. Inhibition of phagolysosome fusion is an important mechanism for pathogenic mycobacteria to avoid killing by macrophages, and AZP treatment did increase co-localization of mycobacteria containing phagosome with lysosome. We hypothesize that these proteins may be endocytosed and then targeted directly to the maturation-arrested mycobacterial phagosome where the endocytosed proteins facilitate killing of the bacteria. Subsequently, the dead bacteria-containing phagosomes fuse with lysosomes. Indeed, we found that alexa fluor-labeled lysozyme was taken up to the endosomal compartment and localized with *M. smegmatis*. Another possible explanation could be that mycobacteria infection alters the host cell membrane resulting in exposure of negatively charged molecules that result in increase in binding of cationic core region of peptide facilitating the permeation of the peptide to reach its intracellular target.

Interestingly, when we compared the mycobactericidal activity of purified lysozyme and elastase, we found important differences. A higher concentration of lysozyme was needed to increase intracellular killing of mycobacteria in THP-1 cells than was needed for direct killing of mycobacteria *in vitro*. The reverse was true for elastase. This difference may either be due to the fact that elastase is more avidly taken up by the cells than lysozyme or that there is a greater synergistic effect of the mycobactericidal effect of elastase and other phagosomal components than is the case for lysozyme. The other possible explanation could be neutrophil elastase activate macrophages through TLR-4 [47] and induce microbicidal activity. Therapeutically, compounds are needed that kills surviving mycobacteria inside the macrophages. The data with lysozyme and elastase indicate that it is not enough to test the direct mycobactericidal activity of potential therapeutic compound it need to be supplemented with data how there compounds increase intracellular killing of mycobacteria in macrophages.

In summary, the current study provides evidence that neutrophils account as one of the innate anti-mycobacterial components in which azurophil granular proteins are more active against mycobacteria, ensuring initial protection from invading bacteria. Addition or induction of azurophilic proteins increases killing efficiency of macrophages by means of direct action or alternatively by facilitating ability of mycobacterial phagosomes to fuse with lysosomes.

## Materials and Methods

### Ethic statement

All research was approved by the Institutional Review Boards (IRBs) of the KIIT University, Lund University and the University of Copenhagen. Written informed consent was provided by the study participants.

### Bacteria, cell lines and monocyte-derived macrophages

*Mycobacterium smegmatis* mc2155 and *M. smegmatis*-GFP were grown in Middlebrook's 7H9 broth medium (Difco) containing 0.05% Tween 80, 0.5% glucose and 0.5% albumin at 37°C on a shaker at 120 r.p.m. *M. bovis* BCG-GFP Pasteur strain (ATCC35734) and *M. tuberculosis* H37Rv were grown in Middlebrook's 7H9 supplemented with OADC (Oleic acid-albumin-dextrose-catalase) and 0.05% tween 80 at 37°C under static condition. GFP expression was maintained by addition of 50 µg/ml hygromycin to the medium.

The THP-1 monocytic cells [17] were maintained at 37°C in 5% CO_2_ in RPMI-1640 supplemented with 10% heat inactivated fetal bovine serum (FBS, Gibco), 10 mM HEPES (Gibco), 1 mM sodium pyruvate, 2 mM L-glutamate, and pencillin- streptomycin solution (Gibco). The cells were seeded onto 24-well culture dishes at a density of 5×10^5^ cells/ml and treated overnight with 20 nM phorbol myristate acetate (PMA) from Sigma to differentiate THP-1 monocyte cells to macrophages. Cells were then washed three times with PBS and incubated for one more day before performing the experiment. Before infection, the cells were washed with 1× PBS and grown in RPMI-1640 medium containing 5% FBS, 2 mM L-glutamate and without penicillin-streptomycin. Mouse macrophage RAW 264.7 cells [17], [22] were maintained in DMEM media (Gibco) supplemented with 10% FBS, 10 mM HEPES, 1 mM sodium pyruvate, 2 mM L-glutamate and pencillin- streptomycin solution at 37°C in 5% CO_2_. To prepare monocyte-derived macrophages, PBMCs were isolated from the buffy coats obtained following informed consent from several healthy human donors by density gradient centrifugation using lymphoprep (Axis-Sheild PoCAS, Oslo, Norway). Purified monocytes were allowed to adhere for 6 days on 6-well plates in the presence of RPMI medium supplemented with 5% human serum and GM-CSF (10 ng/ml).

### Isolation of PMNs and disruption of neutrophils by nitrogen cavitation

PMNs were isolated from blood of healthy donors by dextran sedimentation method followed by density centrifugation as described previously [48]. Briefly, the blood was subjected to dextran sedimentation and the leukocyte rich supernatant was separated by density gradient centrifugation at 400 g for 30 min at 4°C using lymphoprep solution. The pellet was suspended in ice-cold water for 30 seconds to lyse the erythrocytes and the tonicity was restored by addition of equal volume of 1.8% saline. The sample was centrifuged and resuspended in 0.9% NaCl to a concentration of 1×10^6^ cells/ml. To inhibit proteolysis isolated neutrophils were incubated with 5 mM diisopropyl flurophosphate (DFP) (Sigma Aldrich, Milwaukee, WI, USA) for 5 min on ice and centrifuged at 200 g for 5 min. Neutrophils were disrupted by rapid decompression of nitrogen to a pressure of 500–600 psi for 5 min in a nitrogen bomb (Parr instruments, Illinois, USA). The cavitate was collected drop wise, centrifuged at 400 g for 15 min and the post nuclear supernatant containing the neutrophil granules were collected.

To check the survival of *M. smegmatis* and *M. bovis* BCG, the isolated neutrophils (1×10^6^ cells/ml) were seeded in 24-well tissue culture plates for 1 h and then stimulated with 10 µg/ml LPS (Sigma) for another 1 hour. Neutrophils were infected with mycobacteria at a MOI of 10∶1. The cells were lysed at different time points, serial dilutions of lysed cells were prepared in PBS and plated on 7H10 medium supplemented with OADC. Unstimulated neutrophils (with out LPS) were used as control.

### Subcellular Fractionation

Subcellular fractionation of neutrophils was carried out by density gradient centrifugation on a two layer Percoll gradient (density 1.05/1.12) as described previously [49]. 10 ml of post nuclear supernatant was layered gently, avoiding mixture of the gradients. The gradient was centrifuged at 37,000× g for 30 minutes at 4°C in SS34, fixed angle rotor in a Sorvall RC-5B centrifuge. From the bottom α-band containing azurophilic granules, β-band containing peroxidase-negative and gelatinase granules and the clear cytosol present over the band were collected. Percoll was removed from both α- and β-bands by ultracentrifugation at 100,000× g for 90 min, and the granules were resuspended in 0.9% NaCl and stored at −80°C.

### Depletion of defensins from azurophil granule proteins

The granules were freeze-thawed 3–4 times in 1% Triton X-100 and membranes were pelleted by centrifugation at 4°C. The azurophil granule proteins were subjected to cation exchange chromatography on a MonoS column using ÄKTA-FPLC (Amersham Pharmacia Biotech). Bound proteins were eluted with 1 M NaCl (pH 6.5). Subsequently bound defensins were eluted with 0.1 M NaOH. The defensin-depleted azurophil granule proteins (AZP) were concentrated and buffer changed to 0.9% NaCl using centrifugal filtration unit (Milipore).

### Antimycobacterial assay

Neutrophil proteins were incubated with 4–9×10^5^ mycobacteria in Tris-glucose buffer (10 mM Tris, 5 mM glucose, pH 7.4) supplemented with 0.05% tween 80 in 96-well plate. Colony forming units were assayed by plating five microliters of suitably diluted samples in triplicate in 7H10 plates supplemented with OADC at the indicated time points and *M. smegmatis* were counted after 3 days and *M. bovis* BCG and *M. tuberculosis* colonies after 3–4 weeks. The antimycobacterial activity of AZP in the presence of serum was checked by incubating *M. smegmatis* in presence or absence of 5% human serum with 50 µg/ml AZP in Tris-glucose buffer.

### Gel overlay assay

The antimycobacterial activity of neutrophil proteins was determined by gel overlay assay as described previously [21]. Briefly, *M. smegmatis* at log phase were washed and resuspended in 10 mM NaH_2_PO_4_ (pH 7.4). Bacteria (2.5×10^7^/ml) were added to 12 ml melted underlay agarose (0.3% 7H9 medium, 1% agarose type 1 in 10 mM NaH_2_PO_4_ buffer (pH 7.4) and poured into a square Petri dish. Protein samples in duplicate were electrophoresed on acid urea (AU PAGE) gels in 5% acetic acid at 100 V for 1 h 30 min. One of the AU gels was stained with Coomassie brilliant blue and the other one was washed thrice for 4 min in 10 mM NaH_2_PO_4_ (pH 7.4) and then placed over the bacteria containing underlay gel and incubated for 6 h at 37°C. The AU gel was then removed and overlay agarose (5% 7H9 medium, 1% agarose type 1 in 10 mM NaH_2_PO_4_) was poured and incubated over night at 37°C. Clearing zones were visualized by Coomassie brilliant blue staining.

### Peptide identification

Protein bands from AU PAGE corresponding to the clearing zone formed in the gel overlay assay were cut out and digested with trypsin as described previously [50]. The tryptic fragments were identified by MALDI-TOF/TOF mass spectrometry.

### Transmission electron microscopy

Mycobacteria (2×10^7^) were washed thrice with Tris-glucose buffer and incubated in the same buffer with and without AZP for 6 h (*M.smegmatis* and BCG), 6 and 12 h for *M.tuberculosis* at 37°C. Samples of the mycobacterial suspensions were absorbed on to carbon coated copper grids for 2 min, washed with sterile distilled water and negatively stained with 0.075% uranyle formate [50].The grids were made hydrophilic by glow discharge at low pressure in air. Specimens were observed in a Jeol JEM 1230 electron microscope operated at 60 kV accelerating voltage and the images were recorded by Gatan MultiScan 791 CCD camera.

### Intracellular survival assay

5–10×10^5^ RAW264.7, THP-1 and human monocyte-derived macrophages were infected with mycobacteria at a MOI of 10∶1 in RPMI medium containing 5% FBS without antibiotics. Cells were infected with *M. smegmatis* and BCG for 2 h and 3 h, respectively. Extracellular bacteria were killed by addition of 20 µg/ml gentamycin for 1 h. Infected macrophages were incubated with 50 µg/ml AZP, peroxidase-negative, neutrophil cytosolic, and purified azurophil proteins (HBP, elastase and lysozyme, from Calbiochem) for indicated time points in separate set of experiments. Cells were washed, lysed with 0.5% triton X 100 and plated in 7H10 agar to check the intracellular survival of bacteria. Each experiment was performed three times and in each set of experiment the samples were plated in triplicates.

### Immunofluorescence microscopy

For cellular uptake studies, purified human lysozyme (Sigma) was first labeled with FITC (FITC labeling kit, Calbiochem) as per the manufacturer's instructions. 1×10^5^ THP-1 cells were grown on glass coverslips in a 24-well plate and then incubated with 25 µg/ml FITC labeled lysozyme for 1 hour. Cell were fixed using 4% paraformaldehyde, permeabilized with 0.25% saponin and then stained with primary antibody against LAMP-1 (1∶200) (Santa cruz Biotechnology, Santa Cruz, CA) for 60 min at room temperature followed by incubation with Alexa Fluor 594 secondary antibody (1∶1000) (Invitrogen, UK) for 60 min at room temperature. The images were visualized using inverted fluorescence microscope (Nikon Eclipse TE300 equipped with a Hamamtsu C4742-95 CCD camera). To study the localization of lysozyme and *M. smegmatis*, THP-1 cells (5×10^5^) were infected with *M. megmatis*-GFP (MOI 10) for 1 h. The extracellular bacteria were killed by addition of 20 µg/ml gentamicin for 1 h and then incubated with 100 µg/ml alexa fluor 594 labelled lysozyme for 6 h. Finally the cells were mounted in Prolong anti fade reagent with DAPI and visualized under the fluorescence microscope.

For co-localization studies, THP-1 cells were prepared as described before and incubated with medium containing 1% of fetal calf serum for 3 h. Then, cells were treated with 50 µg/ml AZP for 2 h followed by infection with *M. bovis* BCG-GFP as described above. 3 and 12 h after infection, cells were fixed with 4% PFA for 30 min at 37°C. The cells were washed twice with blocking buffer (5% BSA in 1× PBS) and after permeabilization with blocking buffer supplemented with 0.2% saponin and 100 mM glycin for 10 min, cells were incubated with anti-Lamp1 primary antibody to stain late endosomes and lysosomes and then with Alexa fluor 594-conjugated secondary antibody for 1 h. After 3 washings, the cells were mounted using prolong gold antifade reagent with DAPI (Invitrogen) and analyzed by confocal microscopy.

### MTT Assay

To determine cytotoxic activity of AZP on macrophages, RAW 264.7 grown at a density of 2×10^4^ cells/well were treated with various concentrations of AZP for 24 h. To determine the cell viability, the DMEM medium was removed and cells were treated with 0.1 mg/ml MTT (MP Biomedicals, USA) in DMEM for 4 h at 37°C and 5% CO_2_ in dark. The formazan crystals were dissolved in dissolving buffer (11 g SDS in 50 ml 0.02 M HCl, mixed with 50 ml isopropanol). The absorbance was read at 570 nm using ELISA plate reader (Biotek, Germany) and compared with the untreated control cells.

### Quantitative real-time PCR

THP-1 cells were treated with 50 µg/ml AZP for 24, 48 and 72 h. Total RNA was isolated using Trizol according to the recommendations of the supplier (Invitrogen). The concentration of the RNA was determined spectrophotometrically. cDNA was synthesized from 200 ng purified RNA using iScript cDNA synthesis kit (Biorad) according to the instructions given by the manufacturer. Expression of *Beclin-1*, *Atg-5* and *G3PDH* was analyzed using iQSYBR Green Supermix (Biorad). Amplification was performed at 55°C for 40 cycles in iCycler Thermal cycler (Biorad) and data was analyzed using iCycler iQ Optical system software. The relative expressions of the genes were calculated by calculations based on the real-time PCR efficiencies.

### Autophagy analysis

THP-1 cells were grown on 12 well tissue culture plates. After 24 h, cells were treated with 50 µg/ml AZP for another 24 h. Then the cells were fixed and permeabilized as described before. Cells were then stained with primary antibody against LC-3 (rabbit) at a ratio of 1∶500 (Novus biological) for 60 min, washed to remove the excess primary antibody and incubated 1 h with fluorescently labeled secondary anti rabbit antibody (1∶100). After 3 washes, cells were mounted and visualized using inverted fluorescence microscope (Nikon Eclipse TE300 equipped with a Hamamatsu C4742-95 CCD camera).

### Statistical analysis

Statistically significant differences between groups were determined using the Student's *t* test (two-tailed, equal variances). Significance was referred as ** for P<0.0001 and * for P<0.005.

## Supporting Information

Figure S1 **Exponentially grown** ***M. bovis*** **BCG (1–5×10^5^/ml) was incubated with 5–50 µg/ml AZP for 3 h and CFU assay was performed to check the dose dependent anti-mycobactericidal effect of AZP.** Bacteria grown in buffer only served as control. Data are mean ± SD of three independent experiments. Significance was referred as ** for P<0.0001 and * for P<0.005.
(TIF)

Figure S2 **Chromatograms of AZP (A) and peroxidase negative granules (B).** Proteins were eluted by a NaCl gradient in 50 mM Tris-HCl, pH 7.5. FPLC chromatogram displaying purification of proteins using Mono S columns from AZP (**A**) and peroxidase-negative granule proteins (**B**).
(TIF)

Figure S3 **Uptake of FITC-conjugated lysozyme by THP-1.** THP-1 cells were treated with 25 µg/ml FITC labeled lysozyme for 1 h and stained with antibody against LAMP-1 followed by incubation with Alexa Fluor 594 secondary antibody. The co-localization of lysozyme with LAMP-1 was analyzed using fluorescence microscopy.
(TIF)
