# Supplementary figures and images for: Azurophil Granule Proteins Constitute the Major Mycobactericidal Proteins in Human Neutrophils and Enhance the Killing of Mycobacteria in Macrophages
Source: PLoS One. 2012 Dec 14;7(12):e50345. doi: 10.1371/journal.pone.0050345 (PMC3522671; doi:10.1371/journal.pone.0050345)

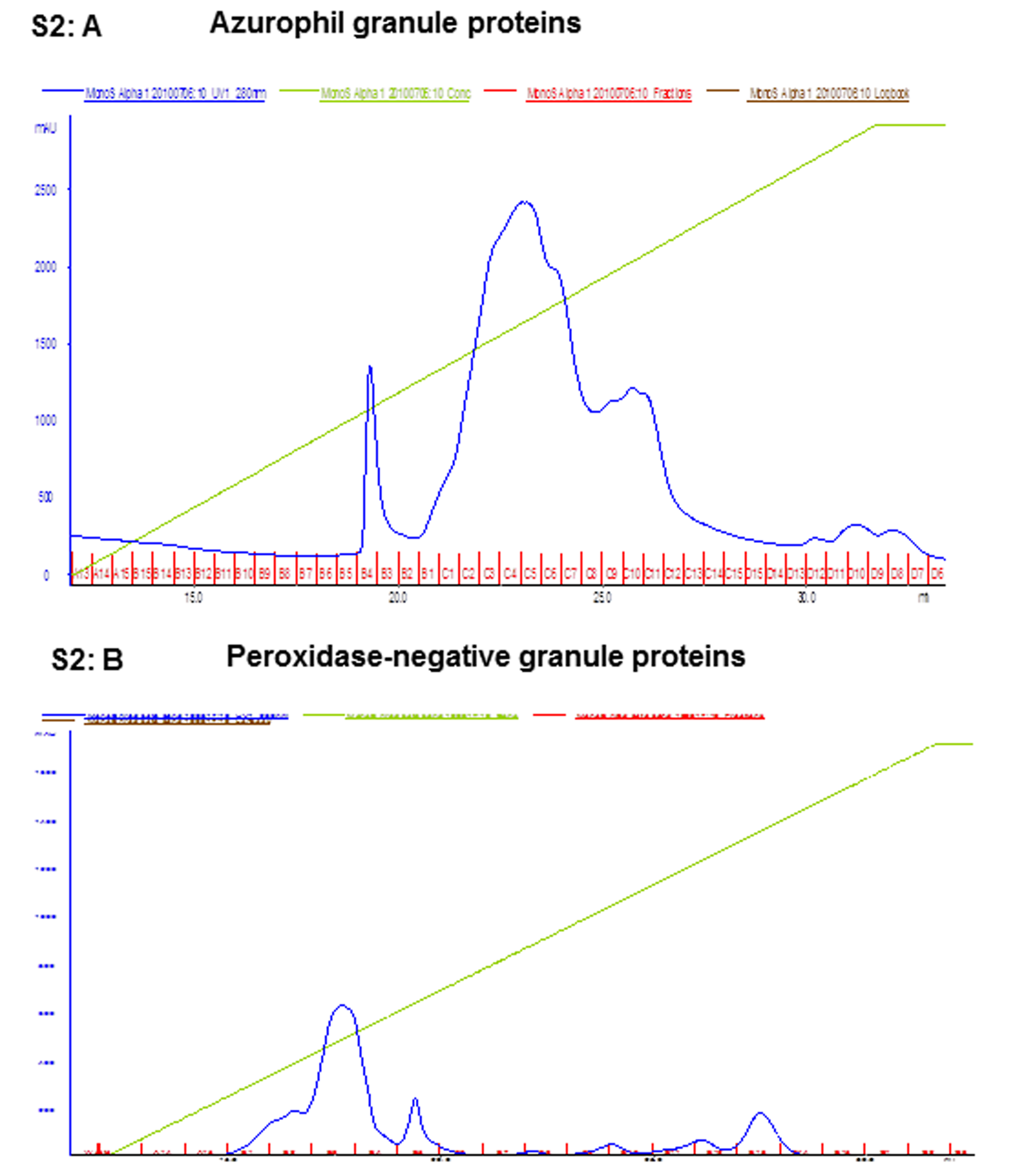

Supplement: Figure S2 — Chromatograms of AZP (A) and peroxidase negative granules (B). Proteins were eluted by a NaCl gradient in 50 mM Tris-HCl, pH 7.5. FPLC chromatogram displaying purification of proteins using Mono S columns from AZP (A) and peroxidase-negative granule proteins (B). (TIF) [file pone.0050345.s002.tif]

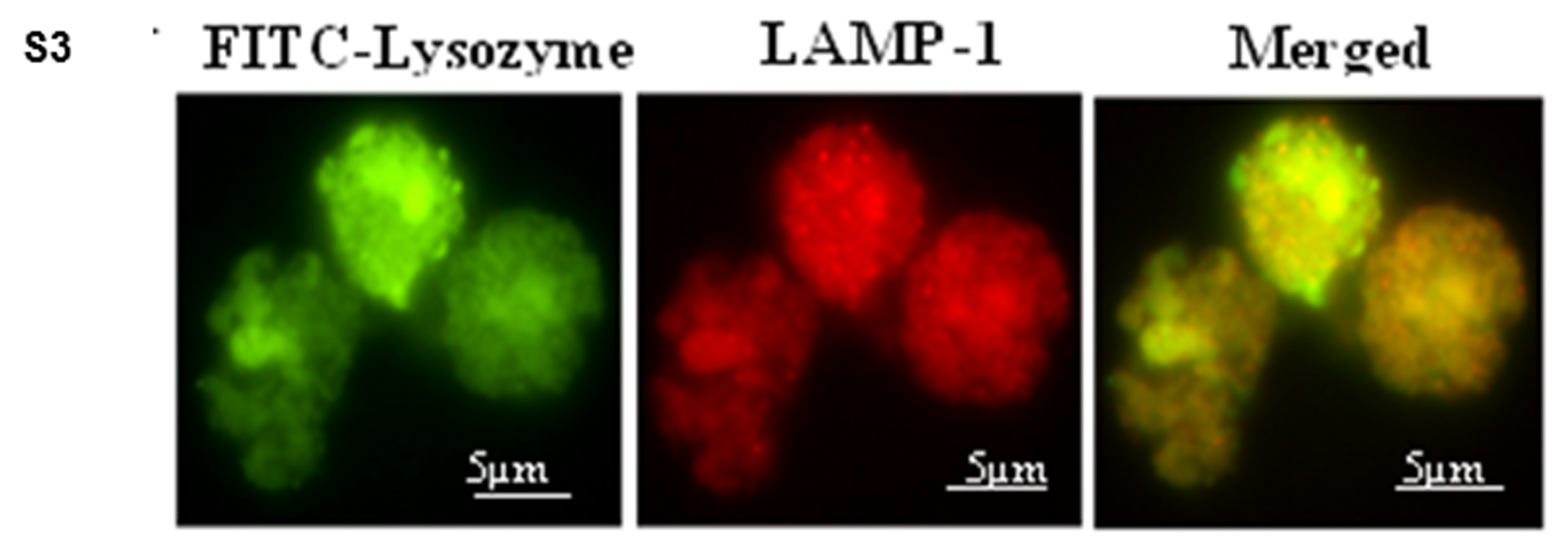

Supplement: Figure S3 — Uptake of FITC-conjugated lysozyme by THP-1. THP-1 cells were treated with 25 µg/ml FITC labeled lysozyme for 1 h and stained with antibody against LAMP-1 followed by incubation with Alexa Fluor 594 secondary antibody. The co-localization of lysozyme with LAMP-1 was analyzed using fluorescence microscopy. (TIF) [file pone.0050345.s003.tif]
